# Safety and performance analysis of acriflavine and methylene blue for *in vivo* imaging of precancerous lesions using fibered confocal fluorescence microscopy (FCFM): an experimental study

**DOI:** 10.1186/s12890-015-0020-4

**Published:** 2015-03-31

**Authors:** Bérengère Obstoy, Mathieu Salaun, Liana Veresezan, Richard Sesboüé, Pierre Bohn, François-Xavier Boland, Luc Thiberville

**Affiliations:** Quant.I.F Litis EA 4108, IRIB, Rouen University, Rouen, F-76000 France; Clinique Pneumologique and CIC INSERM U1404, IRIB, Rouen University Hospital, Rouen, F-76031 France; Department of Pathology, H. Becquerel Cancer Center, Rouen, France; INSERM U-1079, IRIB, Rouen University, Rouen, F-76000 France; Laboratory of Experimental Surgery, EA 3830 GRHV, IRIB, Rouen University, Rouen, F-76000 France

**Keywords:** Diagnosis, Fibered confocal fluorescence microscopy, Genotoxicity, Heller model, Methylene blue, Precancerous lesions

## Abstract

**Background:**

Fibered confocal fluorescence microscopy (FCFM) allows *in vivo* investigation of pulmonary microstructures. However, the bronchial epithelium can only be imaged using exogenous fluorophores. The objective of this study is to compare methylene blue (MB) and acriflavine genotoxicity and to assess FCFM performance for *in vivo* imaging of precancerous lesions.

**Methods:**

Genotoxicity was assessed using the comet assay on both cultured human lymphocytes and NCI-H460 cells, which had been exposed to MB or acriflavine before being illuminated at 660 or 488 nm, respectively. FCFM was performed on precancerous lesions in the hamster cheek pouch model, following topical application of the fluorophores. FCFM data were analyzed according to histology.

**Results:**

No genotoxicity was found using 0.01% (w/v) MB after illumination at 660 nm for 2 and 15 min (5 mW). Acriflavine exposure (0.025%) led to DNA damages, increasing from 2 to 15 min of light exposure at 448 nm in both lymphocytes (83.4 to 88%, p = 0.021) and NCI H460 cell populations (79.9 to 84.6%, p = 0.045). In total, 11 invasive carcinoma, 24 reserve cell hyperplasia, and 17 dysplasia lesions were imaged using FCFM *in vivo*. With both fluorophores, the cellular density increased from hyperplasia to high-grade dysplasia (p < 0.05). With MB, the cellular diameter significantly decreased (48.9 to 13.9 μm) from hyperplasia to carcinoma (p < 0.05). In this model, a cut-off diameter of 30 μm enabled the diagnosis of high-grade lesions with a sensitivity of 94.7% and a specificity of 97%.

**Conclusion:**

Methylene blue can be used safely to image precancerous lesions *in vivo*. This study does not support the use of acriflavine in humans.

## Background

Fibered confocal fluorescence microscopy (FCFM) or probe-based Confocal Endomicroscopy (pCLE) is a recent *in vivo* optical imaging technique that is based on the principle of confocal microscopy. In this technique, the microscope’s objective is replaced by a flexible miniprobe containing 30 000 optical fibers. The probe can be inserted into the working channel of an endoscope and pushed beyond the endoscopic view so as to analyze the distal lung microstructures that come in contact with the tip of the probe. When used in the respiratory tract, this minimally invasive endoscopic technique enables the fluorescence imaging of both proximal bronchi and distal pulmonary interstitial microstructures in humans [1,2].

In humans, elastin is the main constituent of interstitial lung tissue. Elastin excitation at 488 nm wave-length light produces autofluorescence that can be captured by FCFM, and allows the sub-epithelial layer of the bronchial mucosa to be visualized [3]. In a previous study, we demonstrated that the technique makes it possible to analyze the microstructure of the bronchial basement membrane but not the bronchial epithelial cell layer [3]. Consequently, exogenous cellular fluorophores must be employed to observe simultaneously both the epithelial and sub-epithelial layers of the bronchial mucosa using FCFM [4,5].

FCFM studies of the bronchial mucosa have recently been carried out at 488 nm excitation, using either acriflavine, an intercalating DNA element that allows cell nuclei to be imaged [1], or fluorescein, a fluorophore that does not penetrate cells but allows cell interfaces to be visualized [6]. When using a FCFM system at 660 nm, as there is no autofluorescence at 660 nm in the respiratory tract, methylene blue, a fluorophore that temporarily binds to nuclear and mitochondrial DNA, can be used, thus allowing for nuclear and cytoplasmic imaging [4,7].

The majority of epithelial cancers involving the proximal respiratory system, such as squamous cell carcinomas, are believed to be preceded by precancerous lesions [8]. This multi-step carcinogenesis involves different stages, such as hyperplastic lesions, mild/moderate/severe dysplastic lesions, and *in situ* carcinomas. Previous studies have shown that preneoplastic bronchial lesions exhibit a 2-year progression rate of 5% for low-grade dysplasias, 30% for high-grade dysplasias, and 80% for *in situ* carcinomas [9].

The *in vivo* diagnosis of these small-size lesions is currently based on autofluorescence bronchoscopy with targeted biopsies of the areas that display less fluorescence. The main drawback of this endoscopic technique, which is now well-documented, is its low specificity, which is caused by bronchial fluorescence alterations that occur during mucosal inflammation or non-neoplastic hyper-vascularization [10].

We hypothesized that FCFM along with the topical use of fluorophores such as methylene blue or acriflavine would allow the microscopic imaging and diagnosis of the different preneoplastic stages of the respiratory tract lesions, *in vivo, in situ*.

The objectives of this study were:

first, to test whether FCFM with methylene blue or acriflavine allows for diagnosing *in vivo* the different stages of squamous carcinoma development in the experimental cheek pouch model; second, to test the genotoxicity of methylene blue and acriflavine during FCFM.”

## Methods

### Animals

Six weeks-old, 100 g, Syrian golden hamsters (Janvier®, France), were housed in groups of three in pre-sterilized cages placed in a positive/negative ventilation housing system (PNCT3HR30EZ model, Allentown Inc., Allentown, New Jersey, USA). The ambient temperature was 22°C. The circadian rhythm was maintained. The animals had unlimited access to food and water. The study started following 5 days of acclimatization. The animal experiment complied with ethics rules of the Rouen University, and the study protocol was approved by the regional committee for animal ethics (the Normandy ethic committee for experimentation on animals, CENOMEXA; N/03-12-10/25/12).

**Figure 1:**
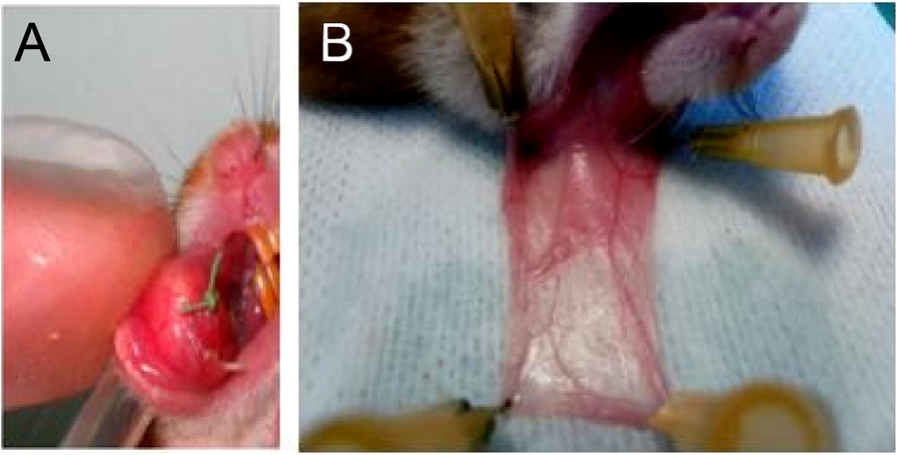
**Hamster cheek pouch. A**/ Implant sutured into the right cheek pouch. **B**/ Everting and spreading a healthy cheek pouch attached to support.

Malignant transformation of the hamster’s right cheek pouch was obtained by suturing an implant impregnated with dimethylbenzanthracene (DMBA) (Sigma Aldrich, St. Louis, USA) according to the technique described by Heller et al. [11]. Figure 1A shows the implant sutured into the jugal mucosa.

The DMBA implant was sutured under general anesthesia using an intra-peritoneal injection of a mixture of ketamine 30 mg/kg (Merial, Lyon, France) and xylazine 10 mg/kg (Bayer, Leverkusen, Germany) following gaseous induction with isoflurane (Abbott, Chicago, USA).

In accordance with the Wani method [12], 4 weeks after the suture of the first DMBA implant, the right cheek pouches were everted and then stained with a solution of arecaidine (Sigma Aldrich, St. Louis, USA) at 1 g/L of mineral oil (Aguettant, Lyon, France) with three applications/week.

### Cells

1. The *in vitro* cells
  Human lymphocytes and NCI-H460 cells (reference HTB-177 American Tissue Cell Collection) were used for the comet assay. The lymphocytes originated from a lymphoblastoid cell line generated from human blood at the INSERM 614 laboratory (Rouen University, France). The cells were cultured in a RPMI 1640 medium containing 10% fetal bovine serum. The cells were protected from light for the entire duration of the experiment.
2. The *in vivo* cells
  The cells were collected by swabbing the cheek pouch, suspended in PBS (1 ml), and centrifuged (700 g, 2 minutes, 4°C). The supernatant was withdrawn, and the cells were then re-suspended in PBS (10^5^/ml, 4°C). At all time, cells were protected from light exposure.

### Fibered confocal fluorescence microscopy

FCFM imaging was carried out with the CellVizio® system (Mauna Kea Technologies, Paris, France). Briefly, the system is made of a laser source, a probe, and a processor. The light bundle was transmitted by the probe, connected to the Laser Scanning Unit (LSU). The probe’s tip was placed in contact with the tissue that was being explored and enabled this tissue to be excited at the LSU's specific wavelength (488 nm or 660 nm). The fluorescent signal emitted by the excited tissue was then collected by the probe, and transmitted to the detector. The system allows to acquire and visualize data as 8–12 images/sec. videos, in real-time.

Two CellVizio® systems were used: one with a LSU emitting at 488 nm, and the other at 660 nm. The power output of the probe when connected to the CellVizio® was 7 mW at 488 nm and 5 mW at 660 nm. The 488 nm source was used for bronchial mucosa imaging with acriflavine, and the 660 nm one was employed for the imaging following local methylene blue application.

Image analysis was performed with the dedicated software MedViewer® 1.1.0. (Mauna Kea Technologies). It allows determining one or two regions of interest (ROI) per lesion, in which cellular density (number of cells/mm^2^) and median cellular diameter were assessed. For each ROI, around 100 cells were analyzed.

### Hamsters’ cheek pouch imaging

Imaging was carried out under general anesthesia (intra-peritoneal injection of ketamine 30 mg/kg and xylazine 10 mg/kg following gaseous induction with isoflurane), directly on the cheek pouch, which was everted, rinsed with sterile water, and spread on a support (Figure 1B).

Imaging of malignant cheek pouches was conducted after 3, 6, 9, 12, 15 and 18 weeks of exposure to DMBA and performed on areas with macroscopic abnormalities.

For each macroscopic lesion identified, at least one microscopic imaging with CellVizio®, one macroscopic photograph, and one histological examination were carried out.

The fluorophores (500 μl), acriflavine (2.5% mass/volume (w/v)) (Sigma Aldrich, St. Louis, USA) or methylene blue (0.1% or 0.01% w/v) (Aguettant, Lyon, France), were directly pipetted onto the cheek pouch and then rinsed with physiological serum (10 ml). The ProFlex CellVizio® probe was placed on the part of the pouch where the fluorophore had been applied. The cheek pouch was imaged in real time. Between four and five recordings of 5 seconds (40 images per recording) were taken of each cheek pouch.

During imaging, biopsies were taken on the exact areas that were imaged with CellVizio®. The animal was then euthanized using an intra-cardiac injection of 1 ml of thiopental (Hospira, Lyon, France), and the entire pouch was removed. The samples were preserved in buffered formalin (4%, Labonord, Templemars, France).

Histological analysis was performed following staining with hematoxylin–eosin–safran (HES). Histological examination of biopsy samples was considered the gold standard.

### The comet assay

The comet laboratory assay protocol was followed (OxiSelect Comet Assay Kit®, Cell Biolabs Inc., San Diego, USA).

The cells (10^5^/ml) were exposed to different experimental conditions and then submitted to an electrophoretic field (300 mA, 1 mV/cm) for 30 minutes at 4°C while being protected from light.

The experimental conditions were:

- application of methylene blue and illumination at 660 nm;
- application of acriflavine and illumination at 488 nm;
- application of fluorophore (methylene blue or acriflavine) without illumination;
- illumination (488 nm or 660 nm) without fluorophore application;
- neither fluorophore application nor illumination (control).

For each experimental condition, different fluorophore contact (2 or 15 minutes) and illumination durations (1 or 2 minutes) were tested. After 2 or 15 minutes, the cells were centrifuged (700 g, 2 minutes, 4°C) and re-suspended in PBS (10^5^ cells/ml, 4°C) twice in order to control the duration of contact between the fluorophore and the cells.

Acriflavine was diluted at 0.025%. Methylene blue was tested at two concentrations: 0.01% and 0.001%.

Following electrophoresis, the cell slides were stained with Vista Green DNA Dye according to the supplier's instructions. The slides were examined using a fluorescence microscope with a FITC filter, and photographs were taken.

The analysis of the images obtained using the microscope was conducted with Image J software (open source, available at http://imagej.nih.gov/ij/). For each cell, the Olive Tail Moment, which reflects the degree of DNA fragmentation, and Tail DNA%, which reflects the percentage of DNA damaged, were determined [13,14].

### Statistical analysis

Statistical analyses were performed using the NCSS 2007 software (NCSS, LLC, Kaysville, USA).

Tail DNA% and Tail Moment were compared using the Mann–Whitney test.

The cell densities and diameters of each histological grade (obtained with CellVizio®) were analyzed using the Kruskal-Wallis test. Cell diameter threshold values for each histological grade were established by Receiver-Operating curves (ROC), when using either acriflavine or methylene blue. A p value <0.05 was considered significant.

## Results

**Figure 2:**
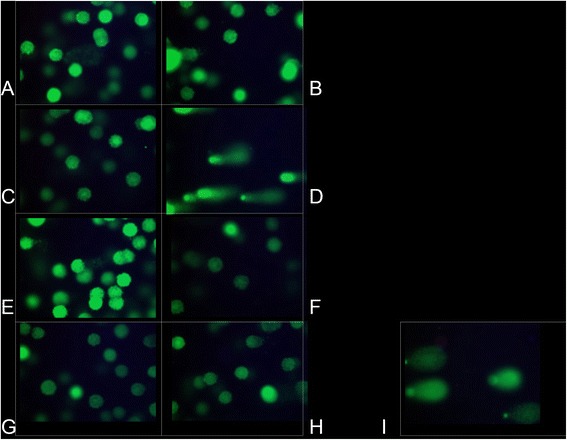
**Comet assay in** ***in vitro*** **and** ***in vivo*** **cells.** Comet assay in NCI-H460 cells not exposed to a fluorophore: **A**/ illumination (2 min.) at 488 nm, **B**/ illumination (2 min.) at 660 nm. Comet assay in lymphocytes: **C**/ acriflavine for 2 minutes, no illumination, **D**/ acriflavine for 2 minutes, then illumination at 488 nm for 2 minutes. Comet assay in lymphocytes incubated with methylene blue: **E**/ 0.001% (2 min.) without illumination, **F**/ 0.01% (15 min.) without illumination, **G**/ 0.001% (2 min.) **with** illumination at 660 nm (2 min.), **H**/ 0.01% (15 min.) **with** illumination at 660 nm (2 min.). Comet Assay in healthy hamster-cheek-pouch cells collected by swab and not put in contact with a fluorophore or illuminated (**I**).
    Genotoxicity of acriflavine
      Figure 2C shows that lymphocytes incubated with acriflavine (0.025%) for 2 minutes, in darkness and not illuminated, did not display any DNA damage (absence of comets).
      Figure 2D shows that lymphocytes incubated with acriflavine (0.025%) for 2 minutes and then illuminated at 488 nm for 2 minutes did display DNA damage. Tail DNA% significantly increased when the contact duration with acriflavine was longer, from 83.4% to 88%, at 2 and 15 minutes, respectively (p = 0.021).
      Regarding the H460 cells, the Figure 3 shows that the Olive Tail Moment parameter significantly increased when the contact duration with acriflavine was extended from 2 to 15 minutes, from 5672.9 ± 1376.6 μm to 7496.9 ± 1959.8 μm (p < 0.001). Similarly, Tail DNA% increased from 79.9% to 84.6% (p = 0.045) when the contact duration with acriflavine was extended from 2 to 15 minutes (data not shown).

1. Genotoxicity assessment
  - Genotoxicity analysis in *in vitro* cells
    Genotoxicity of illumination without fluorophore
      Figure 2 (A-B) shows that a 2-min illumination alone on NCI-H460 cells, at 488 nm (A) and at 660 nm (B), did not induce DNA damage.

**Figure 3:**
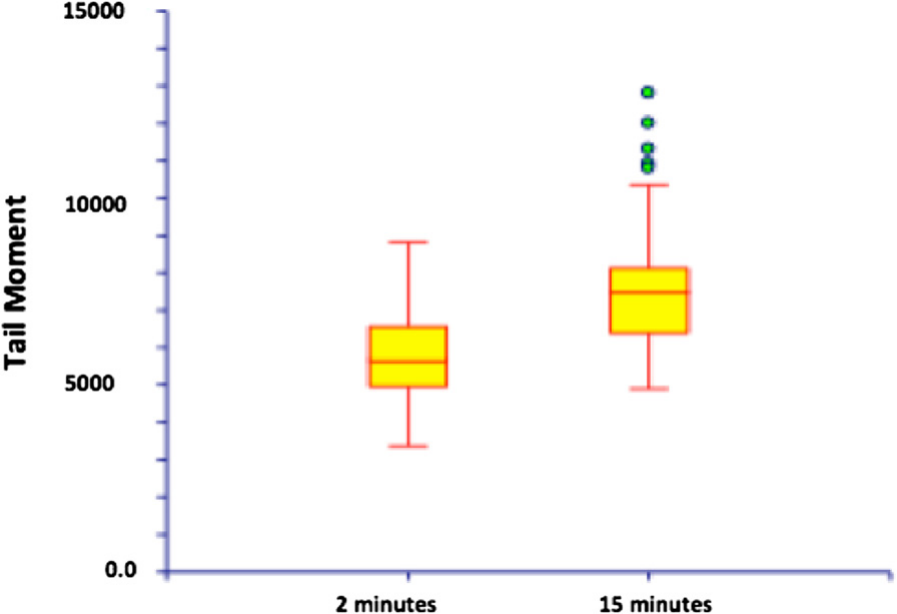
**Variation of DNA damage induced by acriflavine (0.025%) after 2 and 15 minutes of contact with the H460 cells exposed to 2 minutes at 488 nm illumination.**
    Genotoxicity of methylene blue
      Figure 2 (E,F,G,H) shows that the lymphocytes incubated with methylene blue concentrations of 0.001% (E) and 0.01% (F), without illumination, or after illumination at 660 nm for 2 minutes (G) or 15 minutes (H), did not display any DNA damage.
  - Genotoxicity analysis in *in vivo* cells
    This experiment was carried out on 5 animals.
      Figure 2I shows that the healthy cheek pouch cells collected by swabbing displayed DNA alterations at baseline, prior to fluorophore application and before illumination, which were linked to epithelial keratinization. These cells were thus not used for the *in vivo* acriflavine and methylene blue genotoxicity testing.
2. Malignant transformation of the hamster’s cheek pouch
  Overall, 53 animals received an implant impregnated with DMBA. One control animal received a silicone implant without DMBA.
  The implants were well-tolerated, and the animals did not display any local infection. Twenty-eight animals lost their implant early, and new implants were inserted.
  The hamsters did not suffer any weight loss between first implant implementation and the imaging procedure.
  Figure 4 shows the histological lesions obtained.

**Figure 4:**
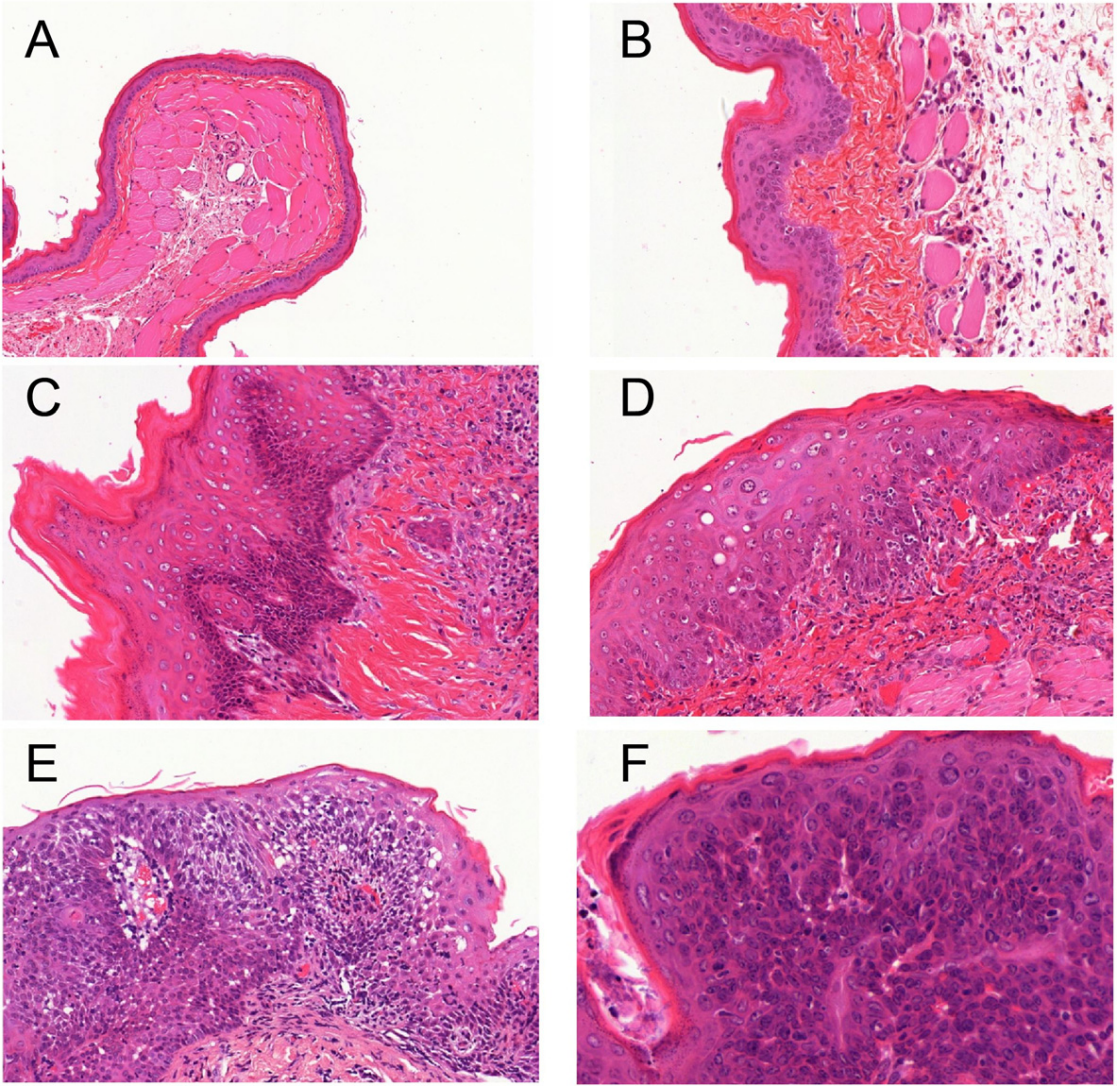
**Histological analysis on hematoxylin-eosin-safran (HES) stained slides: A/ normal epithelium (X2), B/ hyperplasia (X10), C/ mild dysplasia (X10), D/ moderate dysplasia (X10), E/ severe dysplasia (X10), F/ microinvasive carcinoma (X20).**
  Table 1 shows the histological lesions that were obtained and imaged with CellVizio® according to the duration of carcinogen exposure (number of weeks after first implant insertion).

**Table 1:** **Histological lesions obtained and imaged with CellVizio** **®** **according to carcinogen exposure duration (number of weeks after first implant implementation)**

	**>3 weeks**	**>6 weeks**	**>9 weeks**	**>12 weeks**	**>15 weeks**	**>18 weeks**
**Reserve cell hyperplasia**	1	2	3	5	7	6
**Mild dysplasia**	0	2	0	1	1	2
**Moderate dysplasia**	0	0	2	0	2	2
**Severe dysplasia**	0	0	1	0	0	4
**Carcinoma**	0	0	2	0	4	5

**Figure 5:**
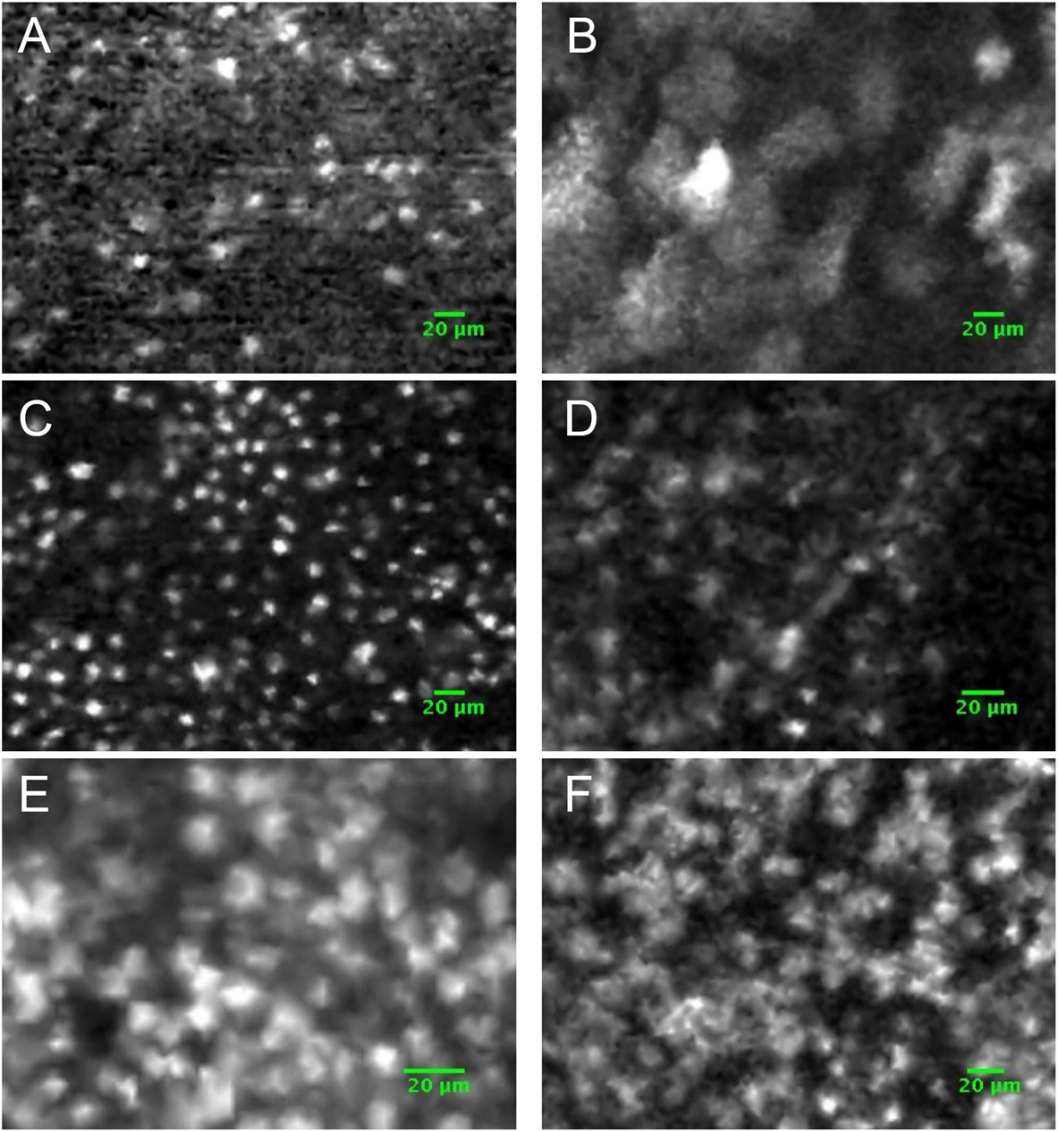
**FCFM imaging of the different stages of carcinogenesis of the hamster cheek pouch after staining with acriflavine or methylene blue. A** and **B**: mild dysplasia, **C** and **D**: severe dysplasia, **E** and **F**: carcinoma.

**Figure 6:**
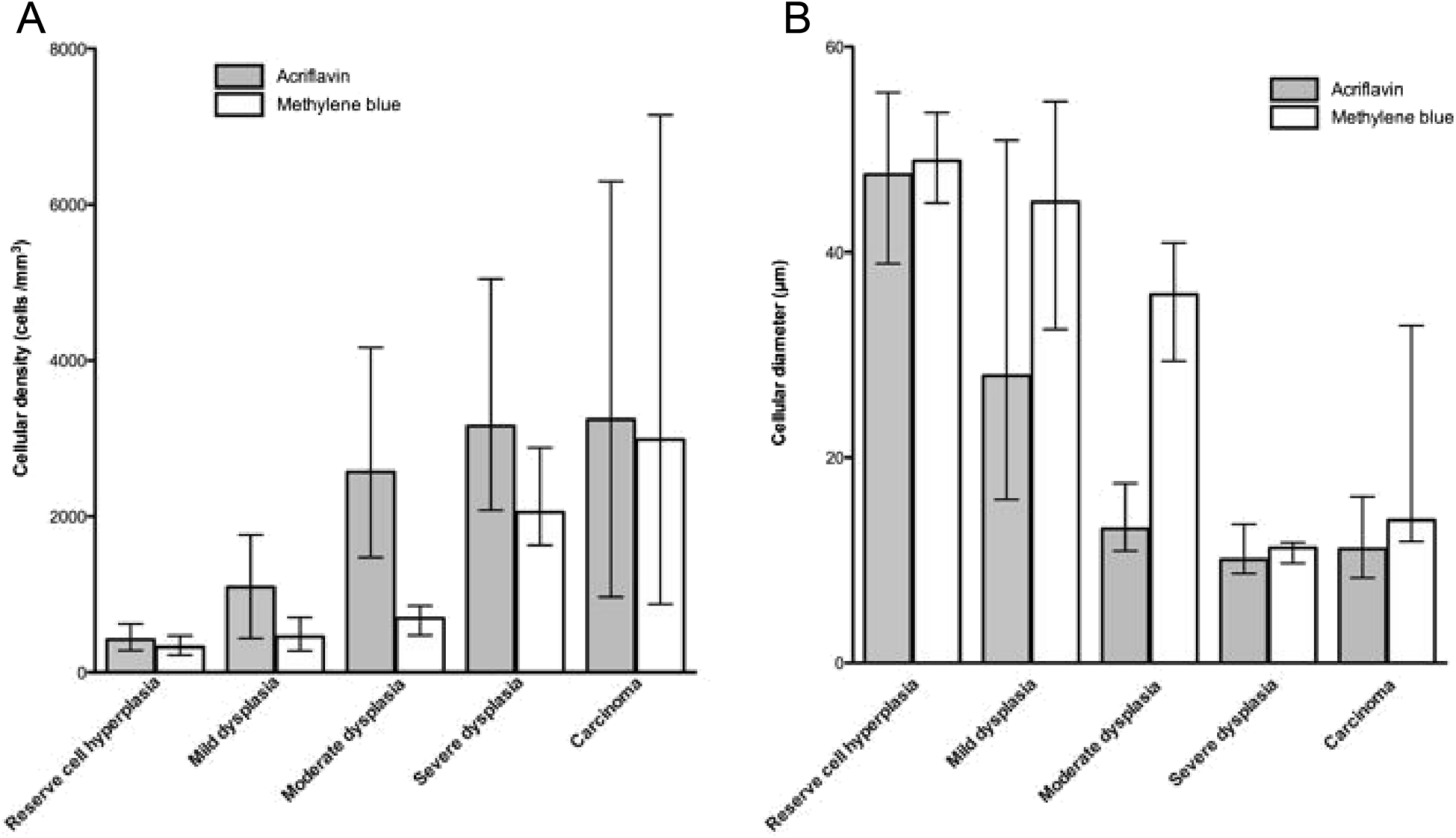
**Change in cell density and diameter using FCFM according to the fluorophore (acriflavine or methylene blue) during the different stages of the malignant transformation of the hamster cheek pouch. A** Median cell density (/mm^2^) calculated from imaging with acriflavine or methylene blue according to histological diagnosis. The data are plotted as median with range. Cell density was significantly higher in acriflavine-aided FCFM compared with methylene blue imaging. Respectively, it was 423 *vs.* 324 cells/mm^2^ in the hyperplastic stage (p = 0.006), 1093 *vs.* 459 cells/mm^2^ in the mild dysplastic stage (p = 0.006), 2574 *vs.* 694 cells/mm^2^ in the moderate dysplastic stage (p < 0.001), and 3163 *vs.* 2055 cells/mm^2^ in the severe dysplastic stage (p = 0.02). **B**. Median diameter (μm) measured using acriflavine or methylene blue-aided FCFM according to histological diagnosis. The data are plotted as median with range. Using acriflavine-aided FCFM, the diameter decreased during the different stages from hyperplasia to high-grade lesions (p = 0.003 between hyperplasia and mild dysplasia, p < 0.001 between mild and moderate dysplasia, p = 0.001 between moderate and severe dysplasia, p = 0.002 between moderate dysplasia and severe dysplasia/carcinoma).
    *FCFM with acriflavine* (Figure 6A): cell density increased significantly, from 423 to 1093cells/mm^2^ between the stages of hyperplasia and mild dysplasia (p < 0.001), and from 1093 to 2574 cells/mm^2^ between the stages of mild dysplasia and moderate dysplasia (p < 0.001).
    *FCFM with MB* (Figure 6A): cell density significantly increased at each stage during progression from hyperplasia to severe dysplasia (p <0.05). Cell density did not differ significantly between severe dysplasia and carcinoma (2055 *vs.* 2989 cells/mm^2^, p = 0.26).
    The cell density was significantly higher with acriflavine compared with MB at the stages of hyperplasia as well as mild, moderate, and severe dysplasia (Figure 6A). In carcinomas, there was no difference in cell density using acriflavine or MB aided -FCFM (3240cells/mm^2^*vs.* 2989cells/mm^2^, p = 0.638).
  - Cell diameter
    The diameters measured with acriflavine aided-FCFM were significantly smaller than those imaged with MB in moderate dysplasia (13 μm *vs*. 36 μm, p < 0.001) and carcinomas (11 μm *vs.* 14 μm, p < 0.001; Figure 6B), as expected because acriflavine mainly binds to nuclei whereas MB diffuses in the cytoplasm. No significant difference was noted for the other stages.
    *In acriflavine-FCFM procedures*, cell diameters decreased from hyperplasia to severe dysplasia and carcinoma (Figure 6B). However, there was no significant change in diameter between severe dysplasia and carcinoma (10 μm *vs.* 11 μm, p = 0.366).
    Using ROC curve analysis, a diameter threshold <13 μm provided a 88.9% sensitivity and 88.9% specificity for the diagnosis of severe dysplasia and carcinoma using acriflavine-FCFM (Figure 7A).

This table shows that the number of lesion increases over time exposure (1 lesion at 3 weeks and 19 lesions after 18 weeks), and that the number of severe lesions also increases over time exposure (none at 3 weeks and 4 severe dysplasia and 5 carcinomas after 18 weeks).
  In total, 11 carcinoma lesions (7 invasive cancers and 4 *in situ* cancers), 5 severe dysplastic lesions, 6 moderate dysplastic lesions, 6 mild dysplastic lesions, 24 hyperplastic lesions, 3 granulomatous lesions, and 3 papillomas were observed.
  The control animal that received a silicone implant without DMBA did not present any macroscopic modification, and histological examination showed no abnormalities.
3. Fibered confocal fluorescence microimaging of cheek pouches during carcinogenesis
  - Cell density
    Examples of FCFM imaging of the cheek pouch at different stage of carcinogenesis are displayed in Figure 5, and cellular features according to histological stage and staining are displayed in Figure 6.

**Figure 7:**
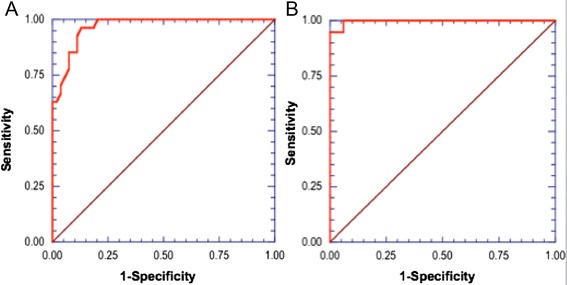
**ROC curves for the diagnosis of high-grade lesions, using acriflavine (7A) and methylene blue (7B). A** With acriflavine FCFM, using a threshold cell diameter <13 μm, sensitivity was 88.9%, and specificity was 88.9% for the diagnosis of “severe dysplasia/carcinoma”. **B** With methylene blue FCFM, a threshold cell diameter <30 μm, sensitivity was 94.7%, and specificity 97% for the diagnosis of “severe dysplasia/carcinoma”.
    *In MB-FCFM procedures*, cell diameters significantly decreased, from 45 μm to 36 μm between mild and moderate dysplasia (p = 0.02); from 36 μm to 11 μm between moderate and severe dysplasia (p = 0.002) ; and from 36 μm to 13 μm between moderate dysplasia and severe dysplasia/carcinoma (p < 0.001). However, the cell diameter appeared significantly larger in carcinomas compared to severe dysplasia (14 μm *vs*.11 μm, p = 0.001) (Figure 6B).
    Figure 7B shows the ROC curve for MB-FCFM diagnosis of “severe dysplasia and carcinoma” lesions. With a cell diameter threshold <30 μm, sensitivity was 94.7%, and specificity was 97%.

## Discussion

*In vivo* FCFM is a new tool for respiratory epithelium imaging, which is hampered by the need of an exogenous fluorophore in order to observe the epithelial cell layer [1-5,7]. The development of fluorophores that can be used in humans is therefore a significant advancement. These fluorophores must be both non-irritating and devoid of any short- and long-term toxicity to be used in humans. They should also permit reproducible nuclear and cytoplasmic imaging.

In this experimental study, we have shown that diluted MB did not induce DNA damage when using the illumination conditions of in vivo confocal endomicroscopy (illumination at 660 nm [5 mW] for 1 to 2 minutes). Methylene blue (Methylthioninium chlorure) is available for the treatment of methemoglobinemia. It is provided in ready to use diluted vials, and no effect on health workers is expected if used under standard conditions. MB is widely used in medical studies, intravenously for treatment of methemoglobinemia [15], and topically, especially for the harvesting of lymph in cancer surgery [16].

In contrast, acriflavine (0.025%) induced considerable cellular DNA damage with illumination at 488 nm for 2 minutes. The percentage of DNA damaged (Tail DNA%) was close to 90% after 15 minutes of acriflavine exposure. This result, which had not yet been published, is directly linked to acriflavine’s DNA intercalating properties.

In humans, confocal endomicroscopy imaging using acriflavine has been performed in the digestive [17] and respiratory [1] tract. Fuchs has recently used acriflavine for *in vivo* confocal imaging of normal and diseased respiratory mucosae in humans [1]. The concentration of acriflavine used in this study was 0.05%, and imaging was conducted after 2 minutes of fluorophore exposure. Fuchs’s study showed that acriflavine is useful for differentiating normal and cancerous mucosa tissues. However our study shows that, with a lower concentration compared to Fuchs’ study and with identical illumination exposure duration, a significant genotoxic risk exists with acriflavine use. Furthermore the GHS (Globally Harmonized System) has classified the product H340 – “may cause genetic defects” and H350 – “may cause cancer”. Our comet assay results support this, and acriflavine should not be used in humans, particularly in patients already exposed to respiratory carcinogens.

The absence of genotoxicity with MB is reproducible at a concentration of 0.01%, which enables good-quality epithelial imaging of the cheek mucosa. However, it cannot be ruled out that higher MB concentrations and more intense illumination may cause DNA damage [18-20]. Davies tested the genotoxicity of MB at 0.1% in cultured mammalian cells [18] illuminated with white light for 2 minutes, revealing significant DNA damage. Certain points need to be clarified: first, in the Davies experiment, the cells in contact with MB were not rinsed, meaning that the duration of contact with the fluorophore was not controlled. By contrast, in our study, the duration of contact between cells and MB was controlled by rinsing the cells. Fluorophore concentrations were also different, as the fluorophore was diluted in PBS. When confocal bronchial imaging with MB is used in humans, the bronchial tree is rinsed immediately after the fluorophore has been applied *in situ* [4,7]. This precaution appears to be sufficient for the safe conducting of *in vivo* microconfocal cellular imaging with the CellVizio®.

Second, the illumination intensity used by Davies differed from that used in our experimental protocol. Davies illuminated his cells with “white light” for 2 minutes with an unknown amount of energy. A reduced illumination wavelength of 660 nm was used in our study, delivering 5 mW at the tip of the fibers for 2 minutes. This duration is longer than the illumination produced by CellVizio® during *in vivo* bronchial endomicroscopic exploration, in which imaging can be performed in a few seconds. Therefore, our results allow us to put forward a safe method for endomicroscopic exploration in humans. Using this method, the mucosa where the fluorophore has been applied is immediately rinsed, and the same area is not illuminated for more than 2 minutes during imaging.

One limitation of our study was the impossibility to test the fluorophore genotoxicity in the *in vivo* cheek pouch cells, as the keratinization process in itself induced DNA degradation [21].

In this study, the use of MB provided reproducible FCFM imaging of the precancerous lesions, comparable to acriflavine. However, unlike acriflavine, MB enabled the imaging of both the cellular nucleus and cytoplasm. In addition, *in vivo* FCFM data allowed to differentiate low-grade precancerous lesions from high-grade lesions using two distinct parameters, namely cell “diameter” and cell “density”. FCFM imaging with MB was of great value for the diagnosis of high-grade lesions, severe dysplasia and cancer. Se was 95% and Sp 97%, which at least equals acriflavine FCFM imaging results.

This study was carried out on a significant number of lesions. In line with Heller’s data [11], after 6 to 20 weeks of carcinogen exposure, 11 carcinoma lesions (7 microinvasive and 4 *in situ* carcinomas), 5 severe dysplasia lesions, 6 moderate dysplasia lesions, 6 mild dysplasia lesions, and 24 hyperplasia lesions were obtained.

One point worth mentioning is that apparent cell density was greater with acriflavine than with MB. This difference was significant as regards the precancerous stages of hyperplasia as well as mild, moderate, and severe dysplasia (p <0.05), but was no longer so in carcinomas (p = 0.6).

These variations may be explained by the keratin layer covering the cheek mucosa. This layer may hinder fluorophore penetration into the epithelial cells of the underlying strata and therefore hamper imaging with CellVizio®. From the stage of moderate dysplasia onwards, the keratin layer became thinner, and so the dysplastic epithelial cells could be observed more distinctly. It is possible that MB cannot penetrate this keratin layer as well as acriflavine. In “en face” imaging, MB only permitted superficial imaging. Imaging with acriflavine, on the other hand, allowed the cells of several cellular layers to be counted, which may account for the observed variations in cell density.

Cell “density” significantly increased between the low- and high-grade preneoplastic stages during imaging, whether conducted with acriflavine or MB. Skala made similar findings with the cheek pouch model using a confocal multiphoton table microscope, demonstrating a significant increase in nuclear density from healthy to precancerous cheek pouches [22]. We went further in our *in vivo* study, showing an increase in cell density between the successive carcinogenesis stages using FCFM.

In this study, a threshold diameter for diagnosing high-grade lesions such as severe dysplasia and carcinoma was established: 13 μm for cells imaged with acriflavine, and 30 μm for cells imaged with MB. Particularly, MB-FCFM imaging had a sensitivity of 94.7% and a specificity of 97%, for the *in vivo* diagnosis of “severe dysplasia and carcinoma” lesions using FCFM. These results confirm that nuclear (acriflavine) and cellular (MB) *in vivo* microimaging was useful for the diagnosis of precancerous lesions and early-stage cancers.

Fuchs has recently demonstrated in humans that applying acriflavine to the bronchial mucosa permitted the differentiation of healthy, inflammatory, and neoplastic bronchial mucosae using confocal endomicroscopy [1]. A preliminary study in humans [4] has demonstrated that, when combined with autofluorescence endoscopy, confocal endomicroscopic imaging of the bronchial mucosa was also practicable *in vivo* using MB. These data, as well as the results obtained in this experimental animal study, indicate that it may be possible to use confocal imaging with MB in humans for precancerous and invasive cancer diagnosis without inducing any genotoxic effects. However, since this study was carried in an animal model, the data may not reflect the situation in humans, especially the threshold values.

## Conclusion

Fibered confocal fluorescence microscopy is an effective tool that requires local fluorophore application to image the epithelial layer. The experiment demonstrates at least equal diagnostic power for methylene blue as compared to acriflavine, without being genotoxic in the tested conditions. For that reason, MB should be used rather than acriflavin as a fluorophore for pCLE imaging in future studies. Further investigations are warranted in order to assess the role of this new imaging modality in the early detection and treatment of superficial precancerous and cancerous lesions of the respiratory tract.

## Abbreviations

DMBA: Dimethylbenzanthracene
FCFM: Fibered Confocal Fluorescence Microscopy
LSU: Laser Scanning Unit
MB: Methylene blue
pCLE: probe-based Confocal Laser Endomicroscopy
